# Pain after earthquake

**DOI:** 10.1186/1757-7241-20-43

**Published:** 2012-06-29

**Authors:** Chiara Angeletti, Cristiana Guetti, Roberta Papola, Emiliano Petrucci, Maria Laura Ursini, Alessandra Ciccozzi, Francesca Masi, Maria Rosaria Russo, Salvatore Squarcione, Antonella Paladini, Joseph Pergolizzi, Robert Taylor, Giustino Varrassi, Franco Marinangeli

**Affiliations:** 1 Anaesthesiology, Intensive Care and Pain Medicine, Department of Health Sciences, University of L’Aquila, L’Aquila, Italy; 2 Department of Civil Defense, Clinical Emergency Psychologist, Pisa, Italy; 3 Hygiene and Preventive Medicine, University of L’Aquila, L’Aquila, Italy; 4 Department of Civil Defense, Sanitary Risk Service, Rome, Italy; 5 Department of Medicine, Johns Hopkins University School of Medicine, Baltimore, MD, USA; 6 Department of Anesthesiology, Georgetown University School of Medicine, Washington, DC, USA; 7 Department of Pharmacology, Temple University School of Medicine, Philadelphia, PA, USA; 8 NEMA Research Inc., Naples, FL, USA; 9 ASL Teramo, National Health Care Service, Teramo, Italy

## Abstract

**Introduction:**

On 6 April 2009, at 03:32 local time, an Mw 6.3 earthquake hit the Abruzzi region of central Italy causing widespread damage in the City of L Aquila and its nearby villages. The earthquake caused 308 casualties and over 1,500 injuries, displaced more than 25,000 people and induced significant damage to more than 10,000 buildings in the L'Aquila region.

**Objectives:**

This observational retrospective study evaluated the prevalence and drug treatment of pain in the five weeks following the L'Aquila earthquake (April 6, 2009).

**Methods:**

958 triage documents were analysed for patients pain severity, pain type, and treatment efficacy.

**Results:**

A third of pain patients reported pain with a prevalence of 34.6%. More than half of pain patients reported severe pain (58.8%). Analgesic agents were limited to available drugs: anti-inflammatory agents, paracetamol, and weak opioids. Reduction in verbal numerical pain scores within the first 24 hours after treatment was achieved with the medications at hand. Pain prevalence and characterization exhibited a biphasic pattern with acute pain syndromes owing to trauma occurring in the first 15 days after the earthquake; traumatic pain then decreased and re-surged at around week five, owing to rebuilding efforts. In the second through fourth week, reports of pain occurred mainly owing to relapses of chronic conditions.

**Conclusions:**

This study indicates that pain is prevalent during natural disasters, may exhibit a discernible pattern over the weeks following the event, and current drug treatments in this region may be adequate for emergency situations.

## Introduction

Prevalence of pain and its therapeutic management in emergency medicine, either in prehospital settings or emergency departments (ED) is an interesting topic in clinical research. Available data [1] indicate that healthcare providers are professionally unprepared to adequately treat “pain conditions” during major emergencies as well as in normal rescue conditions [2]. As already suggested for pain clinics, it is also highly recommended to set and strengthen a routine of continuous assessment of pain evaluated as the fifth vital sign in extreme conditions [3].

In the 48–72 hours after a natural disaster, only anecdotal data are available from out-hospital settings [4-6], even fewer information is available about pain treatment in populations surviving natural disasters [7]. Opioid drugs are almost unavailable in the setting of a natural disaster [8]. A major limiting factor for the efficacy of emergency care, however, is opiophobia of healthcare providers [9]. Another crucial reported point is that medical and nursing staffs are devoid of professional training for diagnosing and treating the majority of painful conditions in emergency medicine [10,11].

These cultural limitations contribute to prevent a modern organization of healthcare assistance during catastrophic events and favor the persistence of the dangerous practice of oligoanalgesia in injured patients. Poor indications are provided by guidelines or validated protocols for pain treatment in patient facing massive emergencies; physicians involved in natural disaster are usually guided by their previous experiences in the setting of war medicine or EDs [12-15].

The aims of the present observational retrospective study was to estimate the prevalence of acute pain and to evaluate drugs used for its treatment in prehospital mass emergency setting during a natural disaster, such as an earthquake. The primary objective of this observational retrospective study was to estimate the prevalence of acute pain in 4 Advanced Medical Presidiums (AMPs) during a five weeks period after an earthquake, in addition to the characterization and frequency of pain conditions. The secondary objective was to evaluate the frequency, type, route of administration, and short-term efficacy of drugs available used for pain control. This, to our knowledge, is the first attempt to document quantitatively pain prevalence and treatment efficacy during a natural disaster.

## Study setting

The present study has been performed after the earthquake that destroyed the city of L‘Aquila (Italy) on April 6th 2009 (see Additional file 1). The consequences of this catastrophic event were immediately evident: 309 deaths, more than 1.500 injured, either rescued or transferred within 12–24 hrs and 65.000 evacuated people. Almost half of the people evacuated have been hosted in 170 tent camps organized around the earthquake areas by the Italian National Civil Defense Service and by volunteers. Up to 3.300 tents have been installed within 48 hours after the disaster; by the end of the second week, the number of tents had increased to 5385, and 100 camp kitchens and 40 Advanced Medical Presidiums (AMPs) and sanitary aids for nursing care had been set up [16] (see Additional file 2). Tent camps were localized in seven Mixed Operating Centers (MOC) in the area of L’Aquila (see Additional file 3), where about 7.000 volunteers and one thousand doctors and nurses were operating [16]. The reference hospital of the whole area, St. Salvatore Regional Hospital in L’Aquila, had to be evacuated on the morning of April 6th because of severe structural damages; within 8 hours, up to 250 inpatients had to be transferred by ambulances and air-ambulances. The Civil Hospital was replaced by a camp hospital, which has been operating for three months and then replaced by the modular structure of the Department of Civil Defense, built up for the G8-Summit in L’Aquila (July 2009). Victims, relatives and evacuees have been assisted by one hundred psychologists and psychiatrists in the emergency centers.

### Advanced medical presidiums (AMPs)

AMP is a medical location similar to an emergency room; it is constituted by a light, pneumatic tent-type structure, provided by the Department of Civil Defense, where voluntary staffs of doctors (2–3 MD/day) and nurses (2–3 NURSES/day) were operating (see Additional file 2). The present staff included healthcare providers with different roles who have been assisting up to 2.412 persons on 8–12 hour duty shifts. The organization of multiple AMPs, as pre-hospital care centers, was the immediate response of healthcare providers to the needs of the population within 3–4 hours after the earthquake in the whole area of L’Aquila. Italian Government Guidelines in case of catastrophic events indicate that AMPs should be activated within 3–4 hours and dismissed within 72 hours after the emergency [16]. In the area of L’Aquila, however, AMPs had been working longer than the three days scheduled, as L’Aquila Civil Hospital was inoperative and other camp- or area-hospitals hospitals were insufficient to meet the needs of the population.

### Study design

The study was approved by the local Ethical Committee. This observational, retrospective, study was carried out in 4 AMPs in the MOC area of L’Aquila during the five weeks after the 2009 earthquake in Italy (from April 7^th^ to May 11^th^ 2009). Design and inclusion and exclusion criteria of the study are reported in the flow chart in (see flow-chart 1) Five weeks after the disaster was the time interval included in the study as in this period the population living in tent camps was mostly constituted by persons surviving the earthquake. After this period, most of the residents have been transferred in residential and lodging houses in different cities; meanwhile the number of aid staffs, firefighters, rescue volunteers and workers providing massive support for rubbles removal, rebuilding and clearing of collapsed houses, progressively increased in tent camps.

### Selection of participants

A total of 958 triage records of patients attending for the first time an AMPs in the four tent camps (Bazzano, Onna, Paganica, Tempera-S. Biagio) for a total population of 2.412 persons, including 1.777 civilians and 635 volunteers, have been examined. Data have been extrapolated from the census performed in the population living in the camps in that period.

It should be pointed out that during the first 2–4 days after the earthquake the study sample included also persons from other districts, giving a great variability.

Inclusion criteria and data selection are shown in flow-chart 1. Demographic parameters registered for each patient included name, surname, gender, age, general physical conditions. Site and type of trauma and pain intensity (verbal Numerical Rating Scale, v-NRS, that is patients were verbally requested to rate their pain) have also been reported. Previous and/or concomitant pathological conditions, including allergies or addiction to tobacco, alcohol and actual drugs have been registered. Based on anamnestic data and both general and neurological physical examination, the pathologic condition, in particular pain conditions, a diagnostic hypothesis was formulated and a therapeutic intervention prescribed with the attempt to minimize the risk of drug interactions or adverse effects. Painful conditions related to specific causes such as infectious diseases (pharyngitis, laryngitis, gastroenteritis) have not been considered in the present report. Similarly, the prevalence of internal pathologies was not assessed, as only painful syndromes have been taken into consideration. Logistic centers included: Bazzano, Onna, Paganica, Tempera-S. Biagio (see Additional file 3). Data have been collected by clinicians in a registration folder and recorded in personal database.

### Patient pain assessment

Pain was quantified by an 11-point numeric scale (v-NRS-11): briefly, adult patients were asked to choose one number on a scale from 0 (no pain) to 10 (severe pain) according to pain intensity [17]. Advantages of this method include easy administration and evaluation, multiple response option, no age-related difficulties or educational barriers in using the scale [18]. Verbal NRS-score recorded in the triage register has been reported in the personal database of the physicians on duty in one of the 4 AMPs only when the main symptom was pain. The clinical and diagnostic evaluation of pain based on the Authors’ experience in pain medicine allowed to provide a qualitative and quantitative analysis of painful conditions in patients in the immediate phases after the natural disaster.

### Drugs

Drugs available in the 4 tent-camps were prescribed by physicians and supplied by healthcare providers of AMP to each patient, every day, during the treatment period. Opioids were kept under lock and registered in order to prevent any abuse and provide a correct therapeutic strategy, considering the shortage and difficult supply of drugs. Immediately after the earthquake, indeed, almost all drugstores were unmanageable, roads were closed and the majority of the population who rushed out of their houses was in the need of drugs for chronic treatment.

### Pain management and short-term pain relief

Criteria for pain treatment depended on the clinical characteristics of the painful syndromes and were mostly based on medical history and physical examination either general and neurological. Therapeutic regimens were based on a simplified three steps WHO’ (World Health Organization) pain ladder due to the shortage of medical devices and drugs, in particular opioids [19].

During the first visit, pain intensity was based on the v-NRS scale and reported in the admission registers for all patients included in the study in addition to signs and symptoms of the painful syndromes. For this scope qualitative characteristics of pain were investigated too: stabbing, burning, paresthesias, allodynia and hyperalgesic signs.

This retrospective analysis included all documents of the first admission to AMPs reporting pain localization, a diagnostic hypothesis, v-NRS-score (T0) and treatment, according to the best clinical practice in pain medicine of the Authors.

Some clinical documents reported the v-NRS-score at admission as well as the v-NRS-score after a very short-term treatment; these parameters have been used to evaluate the immediate effect of drugs (T1 = 1-6 hrs after treatment).

To rationalizing the amount of drugs available, a single dose of a rescue drug and daily doses of drugs used for the initial treatment were provided to the patient.

In our database and admission registers we subsequently searched data of the second evaluation in all patients who returned to our AMPs (T2 = 24 or 48 hours) either because the first drug was ineffective or badly tolerated, or because of relapsing of the pain syndrome or shortage of prescribed drugs.

Also in this case, only clinical documents reporting v-NRS-scores and side effects of drugs were considered; the documents lacking these data were excluded from the analysis. (see flow-chart 2) Based on these data, the efficacy of drug treatment has been evaluated.

### Statistics

Data are presented as means and standard deviations, frequencies are provided as percentages. Prevalence of pain was calculated for the overall population in the first aid centers where the observational study has been carried out.

To minimize bias, sampling was performed during each shift and each day of the week. Frequency was expressed as prevalence with confidence intervals, as the study was performed in 10% of AMPs active during emergency in L’Aquila territory (Bazzano, Onna, Paganica, Tempera-S. Biagio). Confidence intervals of the prevalence in a large sample with high variability allow to estimate the frequency of prevalence in the whole population, with a 95% degree of certainty. The 95% confidence interval was obtained with the following formula, where +/− indicate the upper limit (+) and lower limit (−) interval: P ± 1.96 √P(1-P)/N. Differences in pain score was calculated using Friedman Repeated Measures Analysis of Variance on Ranks and Pairwise Multiple Comparison Test (Tukey Test). P value lower than 0.05 was considered statistically significant. Statistical analysis was performed by SigmaStat® 3.11 Copyright © 2004 Systat Software, Inc.

## Results

### Characteristics of the study population

A total of 958 records of patients attending for the first time one of the four AMPs in tent camps (Bazzano, Onna, Paganica, Tempera-S. Biagio) have been examined. Males were 546 (57%), the majority of interventions (575 cases, 60%) have been provided to patients aged between 15 and 64 years.

The osteo-arthro-muscular system was the apparatus most frequently involved, for up to 219 interventions 22.86% (95% CI, 20.3-25.6) due to traumatic fractures, inflammatory diseases, low back pain (LBP) and relapsed chronic low back pain (CLBP), followed by the respiratory, cardiocirculatory system, mild mental dysfunction and gastrointestinal diseases (Table 1).

**Table 1:** Frequencies of clinical presentation divided according to the involved apparatus evaluated during first admission in AMPs during post seismic period (five weeks)

**Systems affected by pathological conditions**	**N° Patients (n = 958)**	**Frequencies (%) [95% CI]**
Osteomuscular system	219	22.86% [20.3-25.6]
Respiratory system	194	20,25% [17.8-23]
Cardiocirculatory system	93	9.71% [7.9-11.7]
Psychological disorders	91	9.5% [7.8-11.5]
Gastrointestinal system	85	8.87% [7.2-10.8]
Central and peripheral nervous system	85	8.87% [7.2-10.8]
Dermatological system	74	7.72%[6.1-9.5]
Ocular pathologies	33	3.44% [2.4-4.7]
Urogenital system	26	2.72% [1.8-3.9]
Metabolic disorders	18	1.88% [1.1-2.9]
Immunitary system	14	1.46% [0.8-2.4]
Odontostomatognatic system	12	1.25% [0.7-2.1]
Abdominal pain	8	0.84% [0.4-1.6]
Oncological pathologies	6	0.63% [0.2-1.3]

Data are presented as percentage [95%, CI].

### Pain prevalence

During the 5 weeks observation period pain was detected in 332 patients, with a prevalence of 34.6% [95% CI, 31.7-37.7]; males were 180 and females 152, with a prevalence of 54.2% (95% CI, 48.8-59.5) and 45.8% (95% CI, 40.5–51.1), respectively.

Pain score evaluated by verbal Numerical Rating Scale (0 = no pain, 10 = the most severe pain), indicated that severe pain (v-NRS score = 7-10) was the most frequent condition, involving 58.8% (95% CI, 53.3-64), with a prevalence of 20.3% (95% CI, 17.9-23) and an average intensity of 8 ± 0.9. (Table 2).

**Table 2:** v-NRS scale score evaluation of pain at first admission in AMPs (T0) divided by intensity, frequencies of presentation of patient reported pain, number of patients, mean and standard deviation of v-NRS at T0, prevalence of pain intensity on AMPs access during period of study

**NRS**	**%**	**N° Patients (n = 332)**	**Mean NRS**	Prevalence* [95%,CI]
0–3 (mild)	3.6 (2–6.2)	12	3 ± 0	1.2% [0.7-2.1]
4–6 (moderate)	37.6 (32.6-43)	125	5.4 ± 0.7	13% [11–15.3]
7–10 (severe)	58.8 (53.3-64)	195	8 ± 0.9	20.3% [17.9-23]

Data are presented as percentage, mean and standard deviation *Prevalence was calculated on first access at AMPs (n = 958) [95%, CI].

Mean pain intensity was 6.8 ± 1.7 (n = 332). Pain relieving interventions have been provided to 41.9% of patients aged 40–64 years, in particular to 84 males and 55 females 1.54 [OR] (95% CI, 0.99-2.4).

Painful conditions most frequently observed were contusions in 19.88% of cases, muscular tension headache, wounds and low back pain as showed in Table 3.

**Table 3:** Pathologies treated due to the painful conditions

**Pathologies**	**N° Patients (n = 332)**	**% (95%, CI)**
Contusion	66	19.88 (15.9-24.5)
Wounds	52	15.66 (12.1-19.9)
Low Back Pain	42	12.65 (9.5-16.6)
Distorsions	15	4.53 (2.7-7.3)
Fractures	13 (1*)	3.92 (2.3-6.6)
Diffused joint/muscular pain	10 (2*)	3 (1.6-5.4)
Gonarthrosis/gonalgia	6	1.81 (0.8-4)
Tendinitis/Carpal tunnel	6	1.81 (0.8-4)
Great joints (hip and shoulder)	5	1.5 (0.6-3.4)
Cervicobrachial pain	4	1.2 (0.4-3)
Primary Headache	53	16 (12.4-20.2)
Relapsed trigeminal neuralgia	2	0.6 (0.1-2.2)
Herpes Zooster (PHN)	6	1.81 (0.8-4)
Burns	4	1.2 (0.4-3)
Solar burns	12	3.6 (2.1-6.2)
Odontodynia	9	2.7 (1.4-5)
Oncologic pain	6 (2*)	1.81 (0.8-4)
Abdominal pain	8	2.41(1.2-4.6)
Chest pain	5	1.5 (0.6-3.4)
Gynecological/pelvic urologic pain	8	2.41(1.2-4.6)

Data are expressed as percentage and 95% of CI (*) localization of pain was noted like Low Back Pain.

### Time-course of pain conditions

The time-course of pain showed a greater number of first access to AMPs in the first week 42% (35.7-48.5), compared to 33% (27.6-39.6) in second week, 26.2% (20.4-32.8) in third week, 34% (26.5-42) in fourth week and 37% (30.2-44.6) in last week of observation.

In 64 cases 19.3% (95% CI, 15.4-24) a relapse of preexisting pain was reported. In particular, this condition involved 38 cases of benign arthro-osteo-muscular pain, 14 cases of headache, 2 cases of essential trigeminal neuralgia and 6 cases of postherpetic neuralgia (PHN).

**Figure 1:**
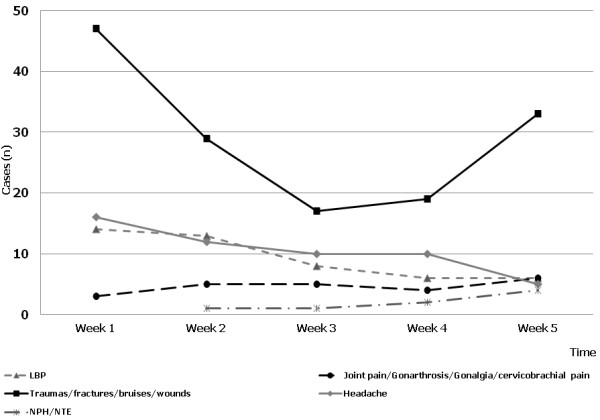
Trend of painful syndromes during the first 5 weeks after the earthquake.

**Figure 2:**
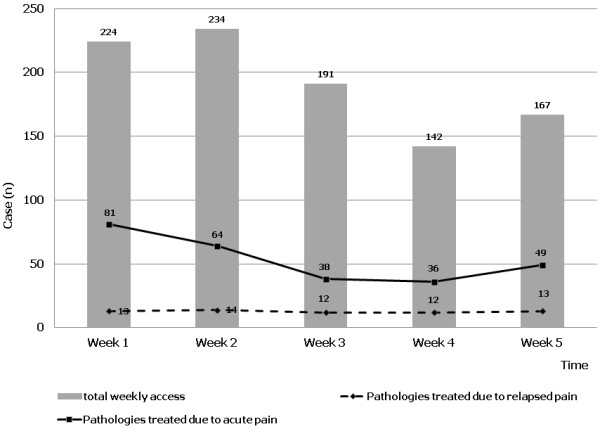
Course over time of pathologies treated due to acute and relapsed pain. Weekly accesses to AMPs, relapsed and acute painful syndromes compared to the total pathological conditions diagnosed.

The trend of the different painful conditions during the 5 weeks following the earthquake is reported in Figure 1. Muscular skeletal traumas and wounds were the most common conditions during the first two weeks and during the last week. Figure 2 shows the time-course of relapsed painful conditions during the 5 weeks of observation. The majority of patients (n = 37) have been examined during week 3, 4, 5 and the remaining 27 patients during week 1 and 2. Figure 2 shows also the number of patients examined during the study; acute pain syndromes, relapsed pain syndromes and other pathologies have been reported for each week.

### Pain medications available during emergency

Pain killers administration was limited to drugs immediately available after the earthquake. All 332 scheduled patient suffering from pain received some medication. In particular, antinflammatory drugs in monotherapy were administered in 24% of cases (diclofenac, ketorolac, nimesulide, ibuprofene, aspirin), paracetamol in 19%. Combination of weak opioids and paracetamol was used in 12% of cases, while weak opioids in monotherapy were preferred in 17% of cases. Strong opioids were administrated in monotherapy in 4%. Combination therapies of strong opioids such as buprenorphine plus pregabalin, acetaminophen plus oxycodone, oral morphine plus ketorolac have been reported in 2% cases. Combination of glucocorticoids plus paracetamol in 4%, antinflammatory combined with muscle-relaxant and adjuvant drugs in 13%, local anaesthetics only in 2% and combined with midazolam in one case were also reported.

A combination therapy of weak opioids plus adjuvants or weak opioid plus NSAIDs have been reported in 1% and 2% cases, respectively.

Other associations, such as butylscopolamine plus tramadol, paracetamol and thiocolchicoside and paracetamol plus amitriptiline, have been reported in 1% cases.

Pain medications have been administered orally in 48% cases (oral solutions, tablets, granules, orally-disintegrating tablet, sublingual tablets), intramuscularly in 26%, intravenously in 24%, and by transdermal route in 2% cases.

Drugs counteracting the adverse effects of pain treatment have been also registered. In 31%, cases H2-blockers have been associated to the infusion of ketorolac, diclofenac and cortisone; metoclopramide has been administered in 22% cases before tramadol and opioids infusion, either by intravenous and oral route, whereas in 3% cases the combined infusion was required. No modification of therapy and no significant side effects were recorded.

### Pain management and relief

**Figure 3:**
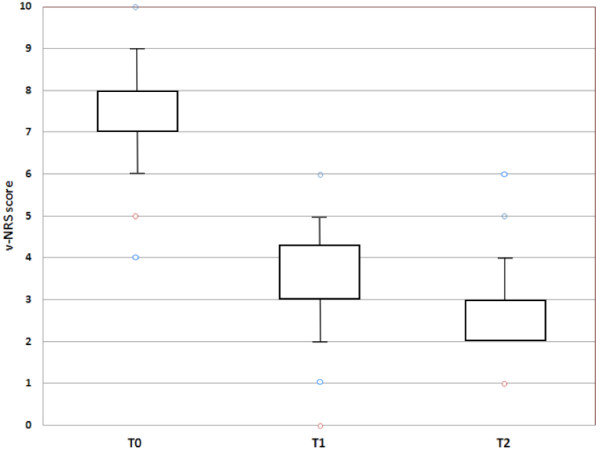
**Box plot of mean v-NRS score at different times T0-T1-T2.** T0: basal v-NRS score upon arrival in the AMPs (n = 181), T1: v-NRS score value after pain treatment (1–6 hr) (n = 181); T2: v-NRS score value after pain treatment (24–48 hr) (n = 137); T0 versus T1 and T2 P < 0.001, T1 versus T2 P < 0.001.

Data from 332 patients initially treated (T0) for pain, in 181 patients v-NRSs within 1 to 6 hours after pain treatment (T1) could be obtained, 54.5% of patients treat for pain relief (Figure 3). Average pain score was 7.59 ± 1.3 before treatment and decreased by 4 v-NRS score points to 3.54 ± 1.2 after treatment (Tukey Test, T0 vs T1, p < 0.001). In 137 out of 181 patients, the analgesic effect has been also assessed within 12–24 hrs after the second administration, 41% of all pain patient treat for painful conditions (T2). Pain score further decreased to 2.78 ± 0.8 (Tukey Test, T0 vs T2 and T2 vs T3, p < 0.001) (Figure 3).

## Discussion

### Pain prevalence, assessment and treatment

The prevalence of acute pain in the population surviving this natural disaster was similar as that reported in pre-hospital settings [20] or EDs [21].

Figures of pain prevalence after earthquakes or natural disasters can be hardly compared due to the large number of variables including population size, city structure, and magnitude of the disaster [22]. Our findings are on line with the report of the 2005 earthquake in Pakistan where up to 29% of the victims experienced some level of pain associated with trauma [23,24]. In rescue conditions acute pain may range from severe pain (es. chest pain) associated with internistic diseases to algic syndromes where pain is the disease, as a distinct nosological entity. Thus, the double characteristic of pain, in itself [25] or comorbid event in multiple organic and psychiatric disorders [26], makes this issue of growing interest in catastrophe medicine [27].

Time-course of prevalence indicates that pain syndromes had the highest value in the first, second and fifth week. Similarly, pain syndromes with different characteristics, namely acute or chronic relapsed, had a biphasic time-course. During the first 15 days after the earthquake most of the acute pain syndromes were caused by traumatic events (fractures, lacerated-contused or incised wounds, joint distortion or dislocation), as a direct or indirect consequence of buildings collapse and nocturnal escape. During week five, pain prevalence increased because of work accidents during rebuilding and removal of rubble from the seismic areas. During week 3 and 4, the prevalence of traumatic cases decreased, but a relapsing of benign arthro-osteo muscular pain and immune reaction diseases, such as postherpetic neuralgia, fibromyalgia, rheumatoid arthritis and trigeminal neuralgia was documented.

The second group of pain syndromes was related to multiple causes, including psychosocial factors; among these causes stressful life conditions, such as living in tents, atmospheric agents, wide range of temperatures, small uncomfortable beds, hard physical work, daily life in emergency centers and unmet personal needs should be considered.

Adverse conditions are known to make the body more susceptible to pathologies, to activate neuroendocrine responses (adaptive, physiological and behavioral) and elicit adaptation processes specific for each person (allostasis) [28]. Pain can be identified as a persistent chronic condition with an emotional impact, becoming in itself a stressing event. Thus, the relationship between stress and pain after a natural disaster is self-perpetuating [29].

This vicious circle may be interrupted by effective painkilling drugs, that prevent chronic pain, particularly in cases of post-traumatic pain, when an aggressive attitude in the administration of strong opioids and ketamine, should be recommended. Two painful conditions with double presentation, acute and relapsed, the primary headache and the low back pain, were pathologies with a considerable prevalence and severity, having a multi-factorial pathogenesis compared to other pain conditions [30,31].

We also observed a higher threshold of pain, particularly in young people tirelessly rescuing the persons buried under rubble during the first few hours following the earthquake. On the other hand, we also reported a reduced threshold of pain, secondary to positive modulators, such as ancestral fear of death, deprivation of sleep, of food and intimacy, mourning, loss of house and social relationships. The influence of mood and mind status on pain perception has been documented by several studies [32], and so it has been demonstrated that antidepressant treatment may have a positive effect on pain [33]. Psycho-cognitive and behavioral disorders reported in the present observational study have been constantly supported by a psychologist in AMPs.

These concepts are essential for understanding analgesic methods during natural disasters.

Severe pain has been reported in more than half of patients; unfortunately, this condition was often underestimated in the management of extrahospital emergency centers [34]. The use of the v-NRS score as a vital parameter allowed us to have an objective evaluation of the problem [6]. Pain scales are helpful for emergency practitioners engaged in disaster medicine after the 2009 earthquake in L’Aquila, as they represent an excellent clinical tool for the evaluation, treatment and follow-up of the patients [35].

In the present study, the most common drugs used for pain treatment included paracetamol, non- steroid antinflammatory drugs and weak opioids administrated in monotherapy in several cases. Pharmacological associations have been frequently used; the synergic mechanism of drugs combination obtained an adequate pain control and minimized individual doses and side effects. The rationale of our approach was to increase the therapeutic plan and drugs combination according to the type and pain intensity; this approach also supplied for the lack of opioid analgesics.

In front of a consistent prevalence of severe pain, strong opioids have been used only in small proportion of cases as monotherapy, in a smaller sample of patients they have been associated with adjuvants and/or NSAIDs; from these figures the need for an increased availability of narcotics drugs during a natural disaster is evident.

Efficacy of pain treatment was documented by a 4 points decrease of v-NRS score, immediately after treatment and at the second assessment. Efficacy and tolerability of drugs was substantially similar among the various therapeutic specialties, either in mono- or multiple-therapy. The high safety and efficacy profile of our simplified pharmacological protocols is probably related to the choice of drugs based on the assessment of pain intensity by v-NRS scale and clinical characteristics of painful conditions [18].

Another therapeutic strategy that we have adopted was assigning prophylactic drugs for the eventual side effects onset that resulted in absence of these events, as happens in the usual schemes adopted in our pain clinical practice.

Shortage of opioids during natural disasters did not only occur in our region, but has been documented in other areas after an earthquake [36]; this aspect is of major concern for the global community. Insufficient administration of strong opioids may depend on difficulties in finding, storing, prescribing and dispensing these drugs. Shortage of painkilling drugs, either opioids and non-opioids, in the first hours after the earthquake was related to difficulties in internal transports and transfer of drugs from and to the hospital pharmacy, which was seriously damaged. Also for these reasons, different forms of pain have been under treated and scarcely controlled, especially in the long-term. In such a disastrous condition, it was important that suffering people deprived from basic healthcare services (including laboratory and x-ray examinations) could at least be treated with major painkilling drugs, including strong opioids.

### Limitations of the study

In the present report, we have not addressed the clinical frames in which the pain was simply an epiphenomenon of conditions that are attributable to specific etiologies such as infectious or inflammatory diseases like pharyngitis, laryngitis or gastroenteritis are. By this point of view, the revision of triage registers has poorly focused on the assessment of the prevalence of internal pathologies, instead taking into consideration only chest pain and gynecological pain. The study it has been limited to just one Italian region with a very small and irregular sample size, due to logistic constraints. The four AMPs involved in this observation also represents only the 10% of the total 40 Advanced Medical Presidiums (AMPs) present in seven Mixed Operating Centers (MOC) in the area of L’Aquila. For these reasons, it is not possible to get a generalization of our data to other natural disasters emergency, but they can represent a good approximation of the clinical good practice on pain medicine in an extreme setting. Pain, in fact, was only documented in one third of the patients, a result that seems rather low; however, this study cannot be correlated with other observations because other studies pain documentation rates with similar results were observed in pre-hospital settings or in EDs, a rather different scenario compared to a post-earthquake setting. In addition, it was also difficult to figure out a global assessment of the effectiveness of pain treatment, because many of the patients were moved and we failed to follow-up as they were transferred to other medical areas or hospitals. Besides, the lack of a strict control of population for logistic causes, represents a possible bias on the trend of reduced pain score, therefore, for this reason, data cannot be generalized in coherent plan of results.

## Conclusions

In a similar catastrophic setting, a trained specialized for pain treatment and emergency medicine staff it becomes essential for succeeding in decreasing the suffering among people, as well as professional suggestions from analogue experiences to be able to reduce mortality and morbidity rates [37]. Results of the present study show that, if there is an adequate training and a specific education in the management of pain for the healthcare providers it might become possible to overcome its enduring emergencies. However, most painful conditions were not strictly related to acute earthquake injuries. In post-natural disaster settings, an accurate use of assessment pain tools and effective treatments may contribute to be facing the most private and human aspects of these events. The pain relief consistently improves the quality of life, mood status, increasingly allowing to sleep and encourage the appetite [38]. All of these factors are known to be positively related to good levels of analgesia. Anaesthetists operating in AMPs reported that administration of appropriate pain killers, like opioids, during the emergencies was rather difficult possibly causing, in fact, a potential global oligoanalgesia, potentially able, in time, to modify the characteristics of pain itself [39]. The majority of patients here studied, reported high pain score but only a small percentage of the analgesics used were opioids. This opiophobia is a common attitude in Italy, as documented by the fact that opioids are scarcely available in extra-hospital settings [40]. The lack of availability of opioid drugs, in this experience, has seriously compromised the possibility to assess a total control over pain. Moreover, we could have had an utterly different approach to cares, if we could have had the access to some of the new formulations of opioids, such as transdermal, sublingual or transmucosal compositions that can facilitate, through expert hands, the administration of therapies, tending more likely to act as fast, appropriate and effective. It is in our opinion that such as pharmaceuticals specialties, would have allowed an easier and more complete management, often in non-invasive way, of the clinical matter, giving part of the solution to a troubled and painful situation caused by this natural disaster [41-44].

In the disastrous situation following an earthquake, an inadequate treatment of pain was the major violation of the psycho-physical integrity of individuals and a severe violation of their rights, as human beings and patients [45]. In fact, the patient, in these particular situations, is a vulnerable person seeking care, and healthcare professionals have the responsibility to provide both physical and psychological care. In this context, the *ars medica* should not just be examining bodies, but it must consider the whole person in his complexity.

## Abbreviations

ED(s), Emergency department(s); AMP(s), Advanced medical presidium(s); MOC, Mixed operating centers; MD, Medical; v-NRS, Verbal numerical rating scale; WHO, World Health Organization; CI, Confidence interval; LBP, Low back pain; CLBP, Chronic low back pain; OR, Odds ratio; PHN, Postherpetic neuralgia; NSAIDs, Nonsteroidal antinflammatory drug(s).

## Competing interests

The authors have no competing interests to declare. No financial support was received to perform this clinical observation.

## Authors’ contributions

AC, GC, PR, PE, UML, MF conceived the study, designed the trial and collected the data; SS, RMR, AC managed the data its quality control and provided statistical advice on study design and analyzed the data; CA, MF, PA, and VG supervised the conduct of the study and the data collection; AC, GC drafted the manuscript and all authors interpreted the data and contributed the contents within the discussion section; TR, PJ and VG reviewed the manuscript; AC, GC, PR, PE, UML, MF takes the responsibility for the paper as a whole. All authors read and approved the final manuscript.

## Supplementary Material

Additional file 1 **“ShakeMap of Central Italy - April 6**^**th**^**2009 (03:32 am)”. **The INGV National Seismic Network recorded an earthquake of magnitude 5.8 (Richter magnitude) (Mw= moment magnitude 6.2) in the area of L’Aquila (central Italy), April 6, 2009 at 3:32 a.m. The epicenter coordinates was: LAT.: 42.33N and LONG. 13.33E, depth at 8.8 km. The earthquake was characterized by an extensional mechanism, with fault planes orientated NW-SE and NE-SW direction of extension. [Font: Istituto Nazionale di Geofisica e Vulcanologia (http://www.ingv.it)]. Click here for file

Additional file 2 “Advanced Medical Presidiums (AMPs)”. Click here for file

Additional file 3 “Tent camps”. Click here for file
