# Supplementary figures and images for: Pain after earthquake
Source: Scand J Trauma Resusc Emerg Med. 2012 Jun 29;20:43. doi: 10.1186/1757-7241-20-43 (PMC3439252; doi:10.1186/1757-7241-20-43)

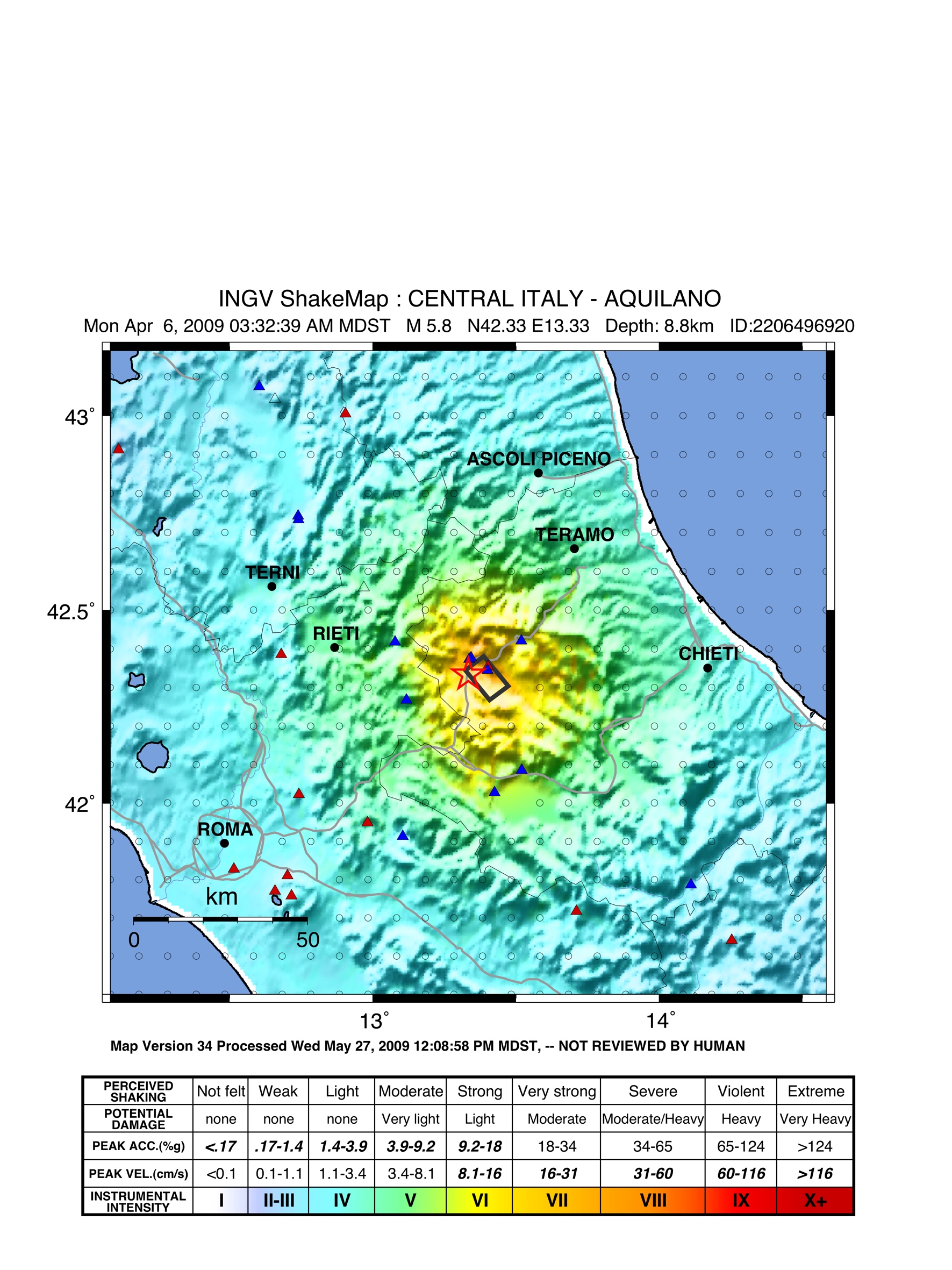

Supplement: Additional file 1 — “ShakeMap of Central Italy - April 6th2009 (03:32 am)”. The INGV National Seismic Network recorded an earthquake of magnitude 5.8 (Richter magnitude) (Mw= moment magnitude 6.2) in the area of L’Aquila (central Italy), April 6, 2009 at 3:32 a.m. The epicenter coordinates was: LAT.: 42.33N and LONG. 13.33E, depth at 8.8 km. The earthquake was characterized by an extensional mechanism, with fault planes orientated NW-SE and NE-SW direction of extension. [Font: Istituto Nazionale di Geofisica e Vulcanologia (http://www.ingv.it)]. [file 1757-7241-20-43-S1.jpeg]

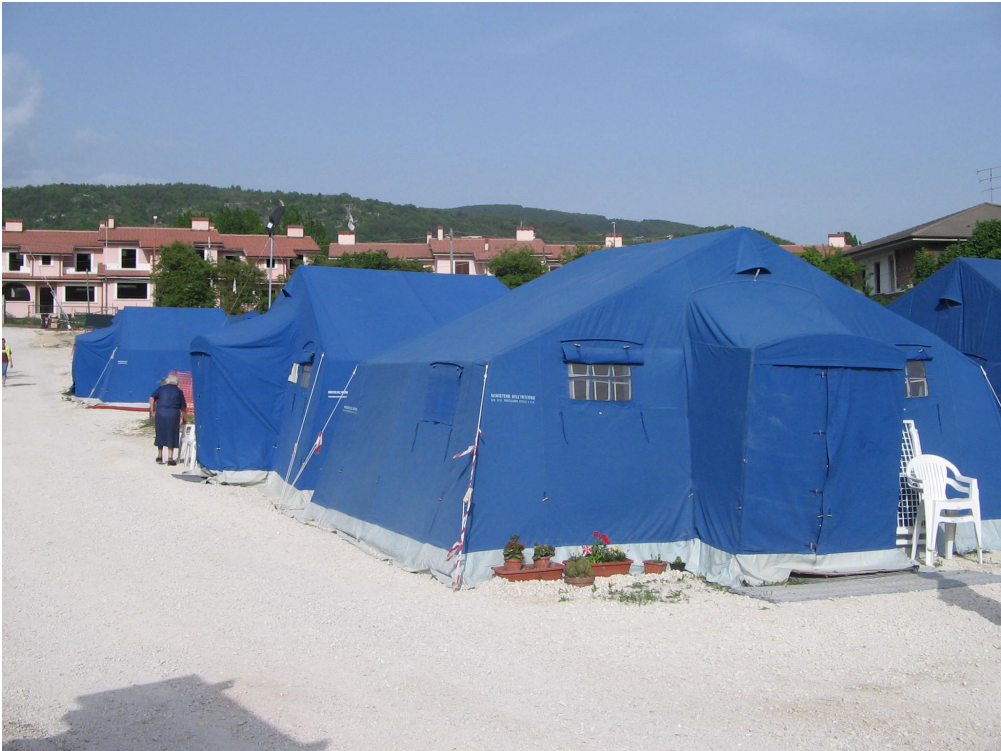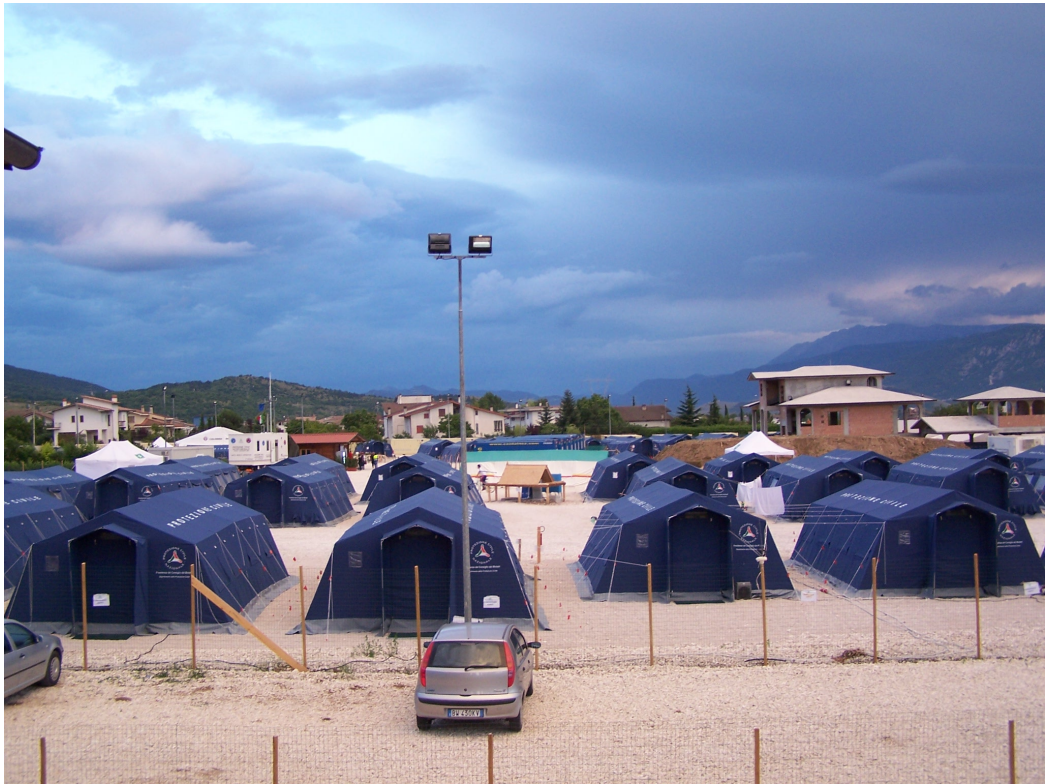

Tent camp of Tempera.

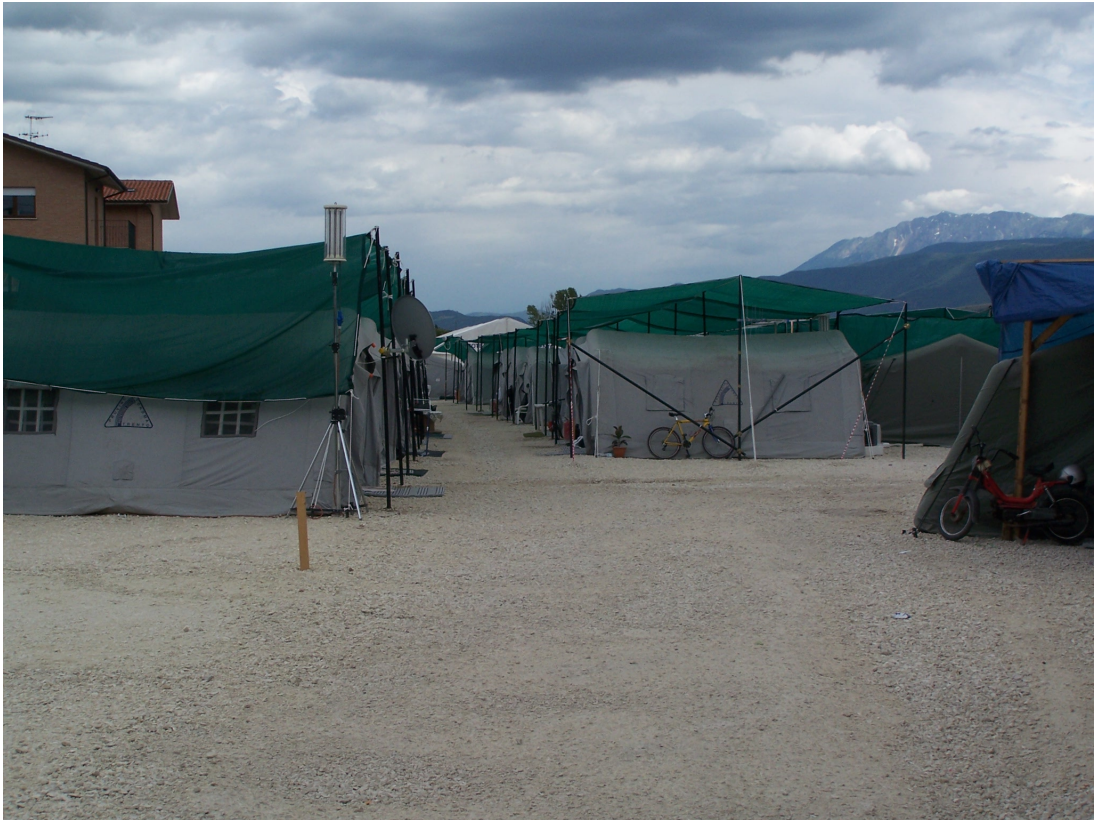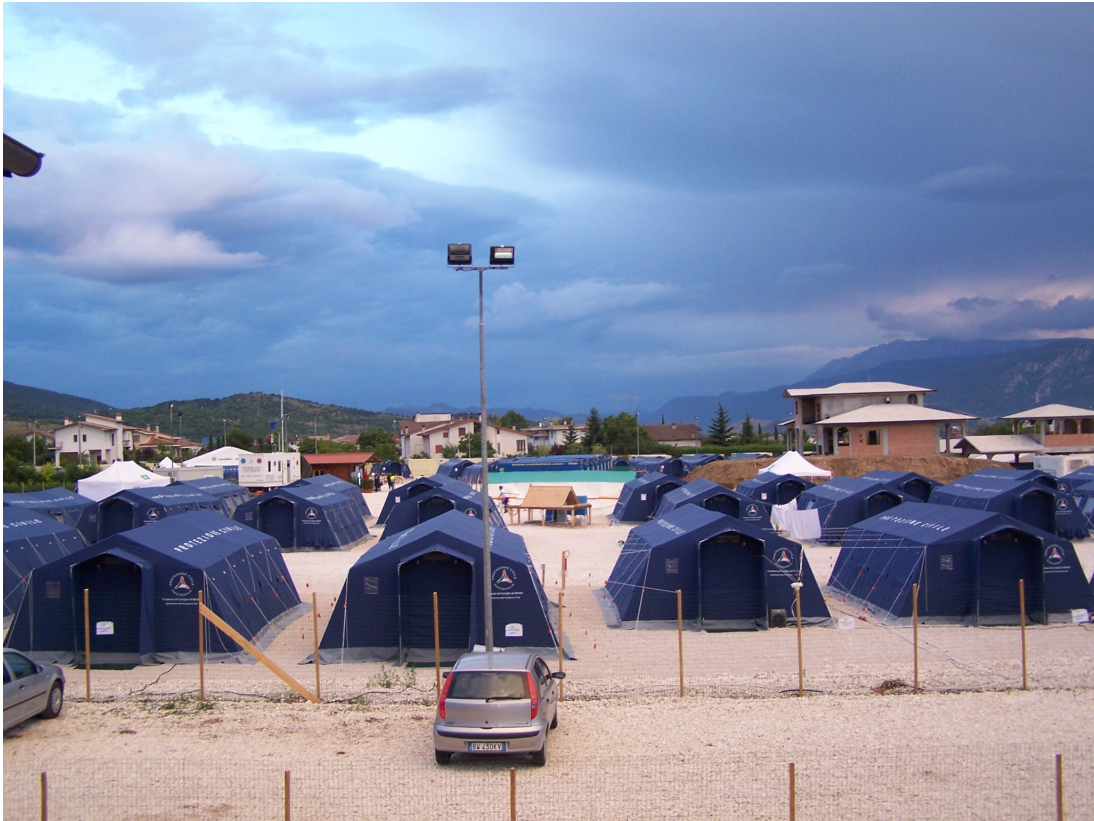

Tent camps of Paganica and Tempera.

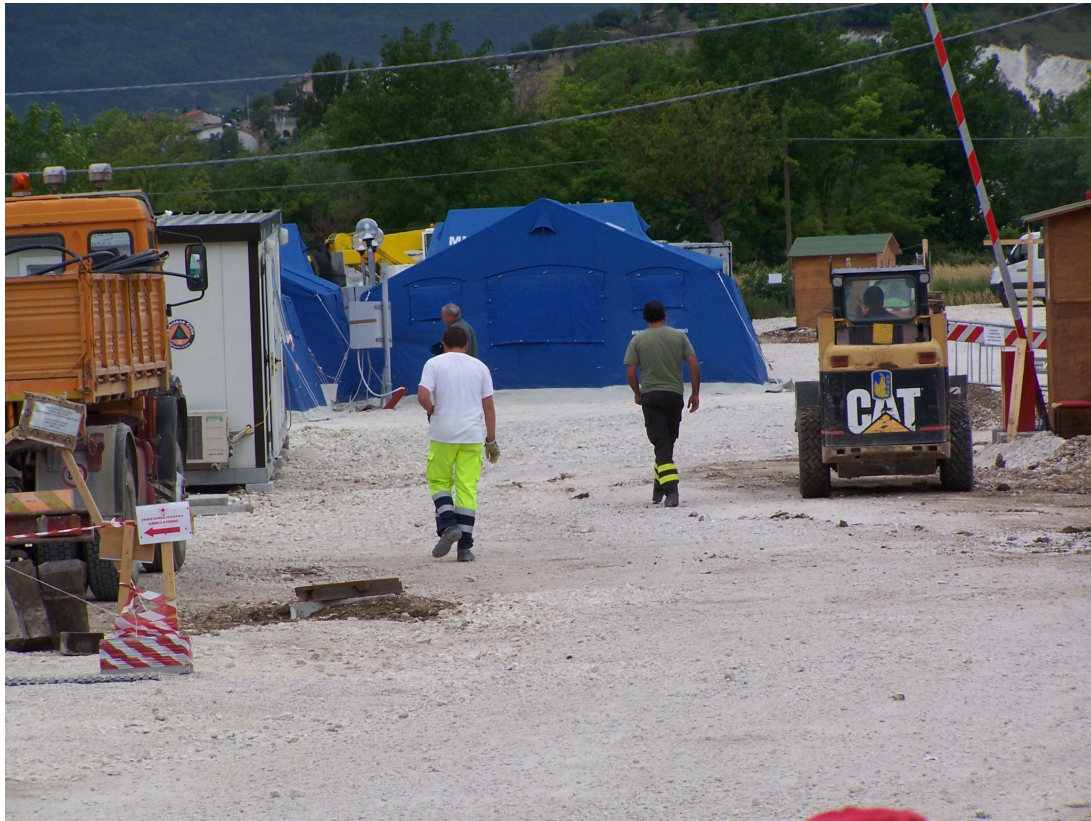

Volunteers at work in tent camp.

Supplement: Additional file 3 — “Tent camps”. [file 1757-7241-20-43-S3.pdf]
