# Supplementary material for: Pain after earthquake
Source: Scand J Trauma Resusc Emerg Med. 2012 Jun 29;20:43. doi: 10.1186/1757-7241-20-43 (PMC3439252; doi:10.1186/1757-7241-20-43)
Supplement: Additional file 2 — “Advanced Medical Presidiums (AMPs)”. [file 1757-7241-20-43-S2.pdf]

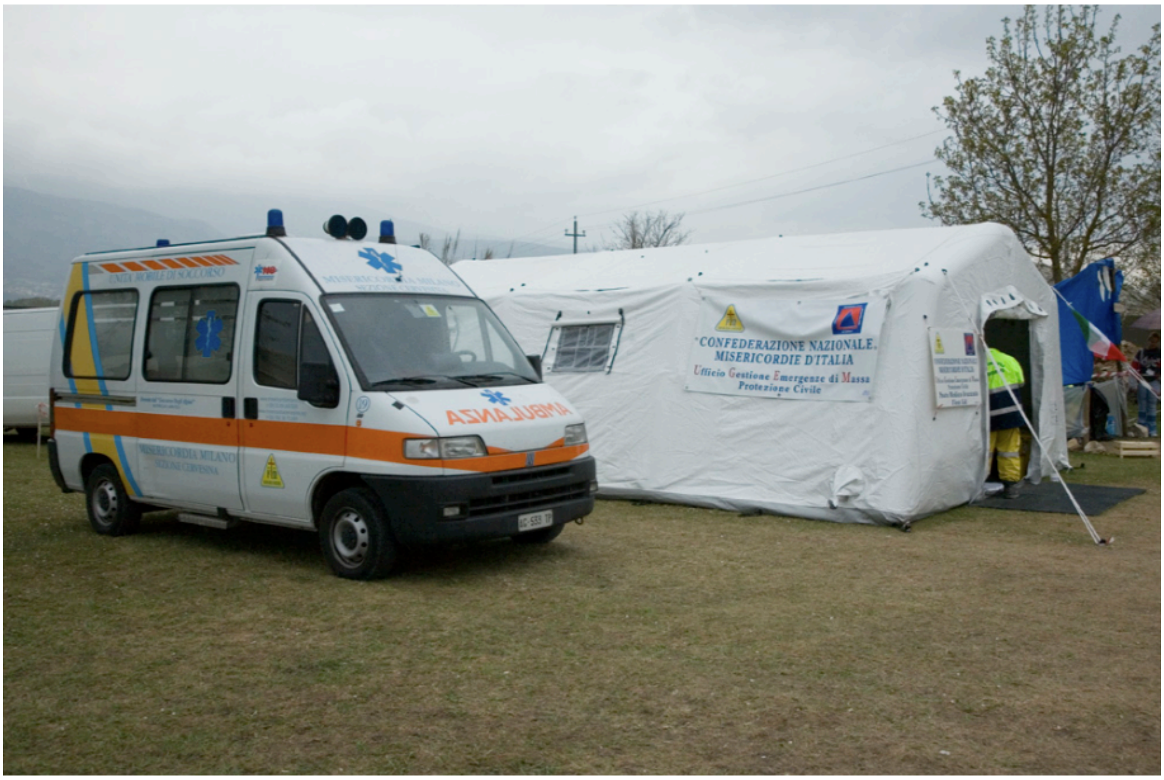

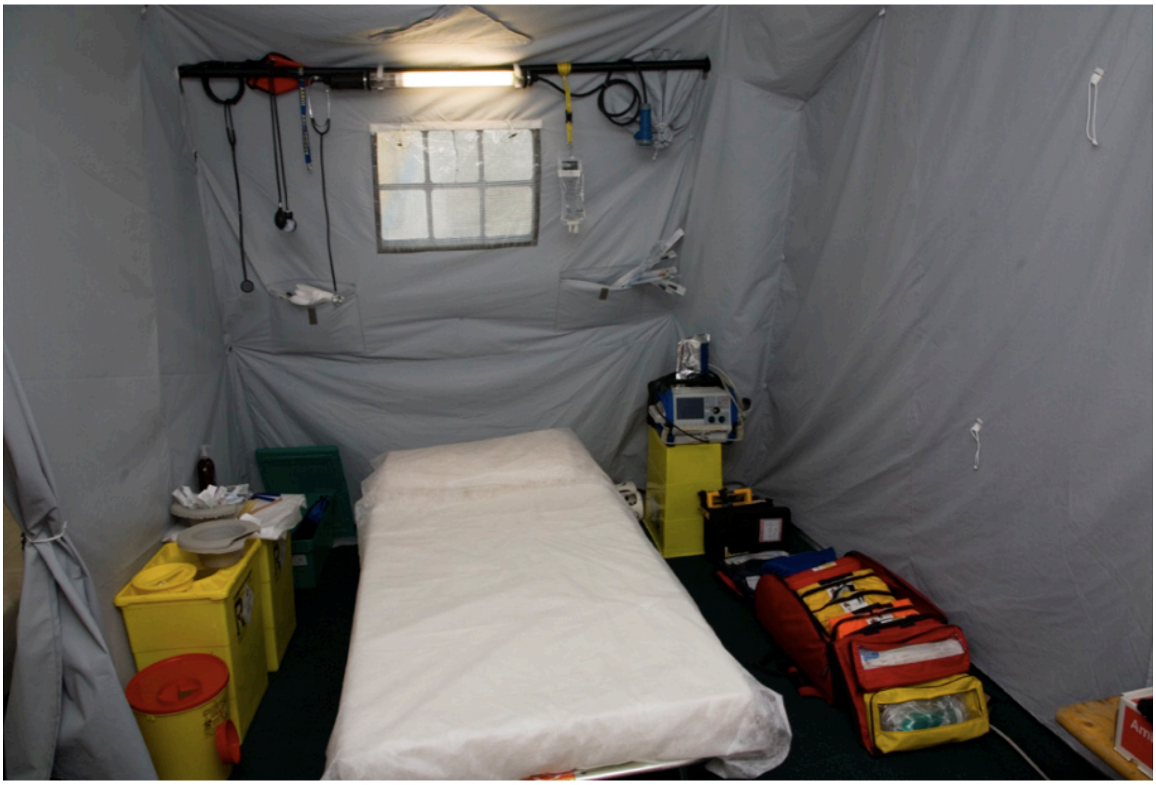

AMP site in Onna tent camp a light, pneumatic tent-type structure.

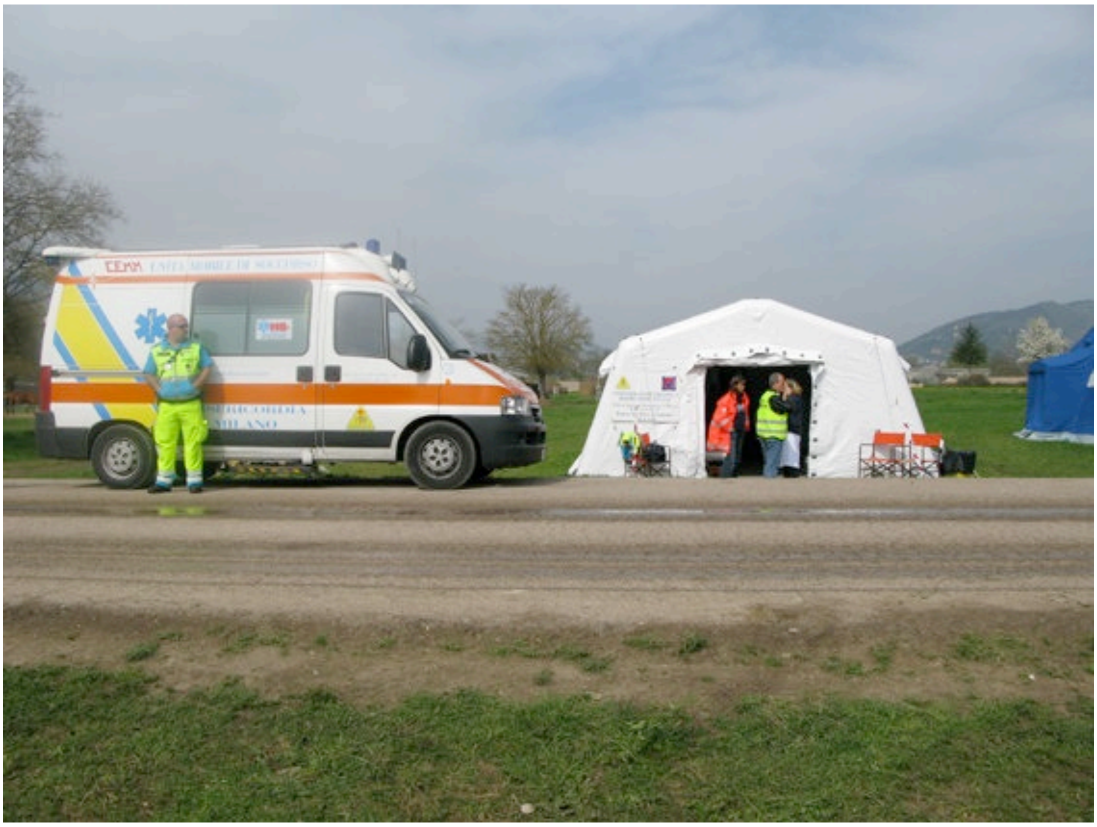

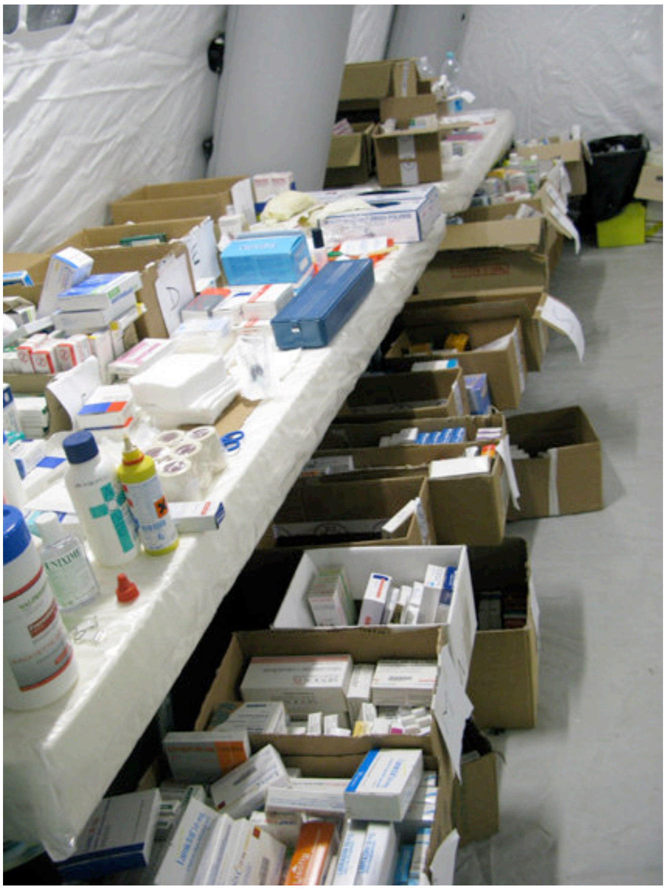

AMP of Onna tent camp, drugs storage inside advanced medical presidium (AMP).

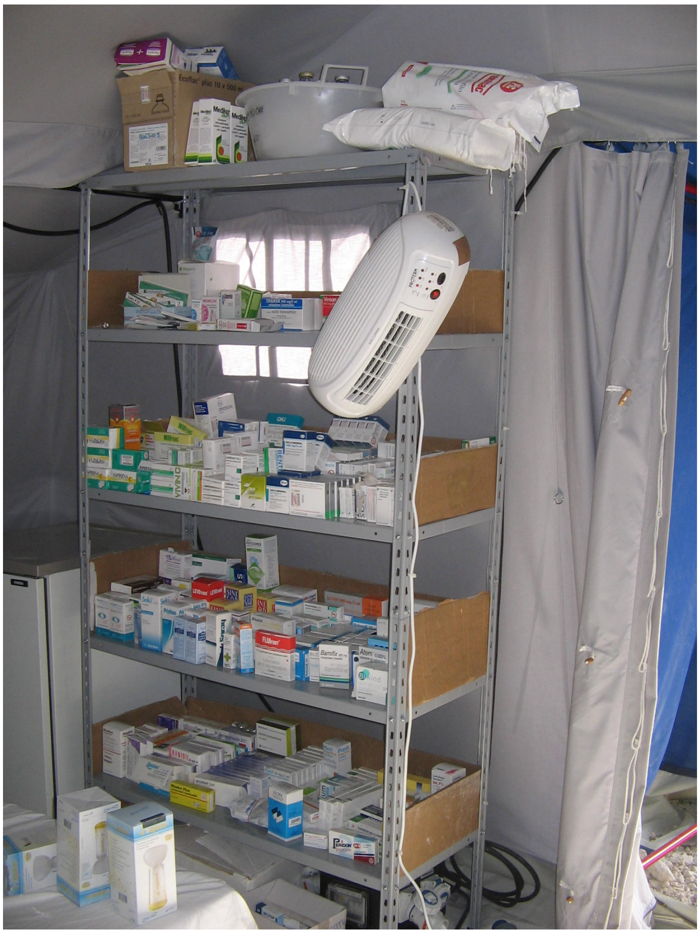

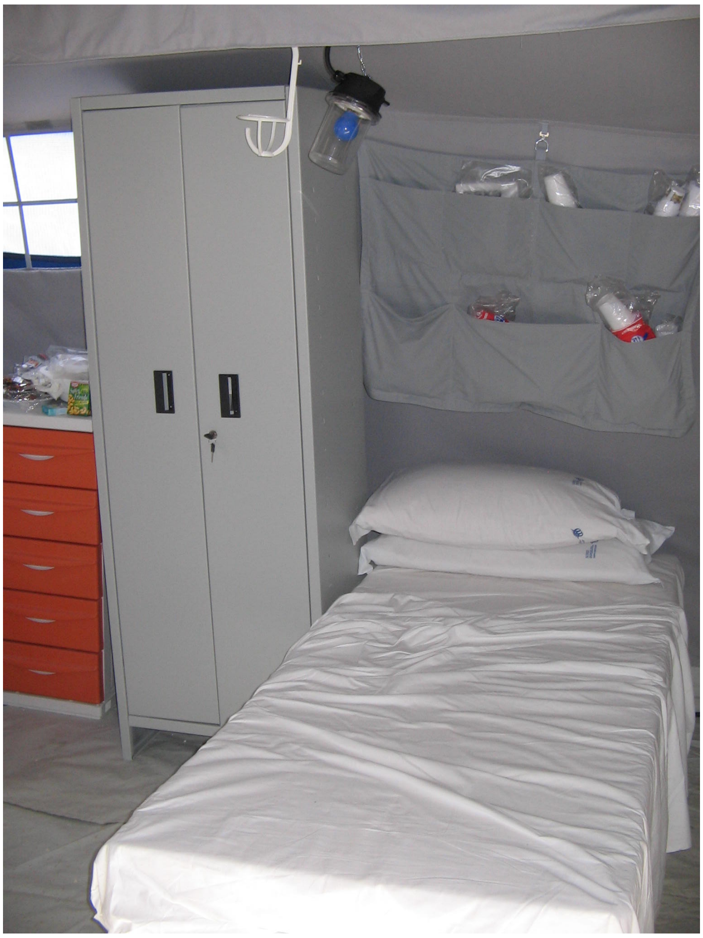

AMP of San Biagio-Tempera.
